# Analysis of Clonal Type-Specific Antibody Reactions in *Toxoplasma gondii* Seropositive Humans from Germany by Peptide-Microarray

**DOI:** 10.1371/journal.pone.0034212

**Published:** 2012-03-28

**Authors:** Pavlo Maksimov, Johannes Zerweck, Aline Maksimov, Andrea Hotop, Uwe Groß, Katrin Spekker, Walter Däubener, Sandra Werdermann, Olaf Niederstrasser, Eckhardt Petri, Marc Mertens, Rainer G. Ulrich, Franz J. Conraths, Gereon Schares

**Affiliations:** 1 Federal Research Institute for Animal Health, Institute of Epidemiology, Friedrich-Loeffler-Institut, Wusterhausen, Germany; 2 JPT Peptide Technologies GmbH, Berlin, Germany; 3 German National Consulting Laboratory for Toxoplasmosis, Department of Medical Microbiology, University Medical Center Göttingen, Göttingen, Germany; 4 Institute of Medical Microbiology and Hospital Hygiene, Heinrich-Heine-University Düsseldorf, Düsseldorf, Germany; 5 Institut für Arbeits und Sozialhygiene Stiftung, Kyritz, Germany; 6 BG Kliniken Bergmannstrost, Halle, Germany; 7 Novartis Vaccines and Diagnostics, Marburg, Germany; 8 Federal Research Institute for Animal Health, Institute for Novel and Emerging Infectious Diseases, Friedrich-Loeffler-Institut, Greifswald - Insel Riems, Germany; Federal University of São Paulo, Brazil

## Abstract

**Background:**

Different clonal types of *Toxoplasma gondii* are thought to be associated with distinct clinical manifestations of infections. Serotyping is a novel technique which may allow to determine the clonal type of *T. gondii* humans are infected with and to extend typing studies to larger populations which include infected but non-diseased individuals.

**Methodology:**

A peptide-microarray test for *T. gondii* serotyping was established with 54 previously published synthetic peptides, which mimic clonal type-specific epitopes. The test was applied to human sera (n = 174) collected from individuals with an acute *T. gondii* infection (n = 21), a latent *T. gondii* infection (n = 53) and from *T. gondii*-seropositive forest workers (n = 100).

**Findings:**

The majority (n = 124; 71%) of all *T. gondii* seropositive human sera showed reactions against synthetic peptides with sequences specific for clonal type II (type II peptides). Type I and type III peptides were recognized by 42% (n = 73) or 16% (n = 28) of the human sera, respectively, while type II–III, type I–III or type I–II peptides were recognized by 49% (n = 85), 36% (n = 62) or 14% (n = 25) of the sera, respectively. Highest reaction intensities were observed with synthetic peptides mimicking type II-specific epitopes. A proportion of the sera (n = 22; 13%) showed no reaction with type-specific peptides. Individuals with acute toxoplasmosis reacted with a statistically significantly higher number of peptides as compared to individuals with latent *T. gondii* infection or seropositive forest workers.

**Conclusions:**

Type II-specific reactions were overrepresented and higher in intensity in the study population, which was in accord with genotyping studies on *T. gondii* oocysts previously conducted in the same area. There were also individuals with type I- or type III-specific reactions. Well-characterized reference sera and further specific peptide markers are needed to establish and to perform future serotyping approaches with higher resolution.

## Introduction

Infection with the intracellular protozoan parasite *Toxoplasma gondii* is often asymptomatic or causes flu-like symptoms in immunocompetent individuals. Primary maternal infection with the parasite during pregnancy may lead to abortion or induce disease in the transplacentally infected fetus. Toxoplasmosis is often fatal in immunocompromised patients [1], [2], [3].

*T. gondii* has a clonal population structure. North America and Europe are dominated by three clonal lineages of *T. gondii*, i.e. the clonal types I, II and III. Type II is most abundant in infected humans and domestic animals [4], [5], [6], [7], [8]. While type III strains are abundant in animals, they are rarely seen in humans [4], [5], [6], [7], [9], but this distribution may be impaired by a sampling bias. Previous studies suggested that type I strains are relatively rare in animals and humans and they have been predominantly found in immunocompromised patients who had experienced a reactivation of *T. gondii* infection, which frequently occurs in HIV-infected toxoplasmosis patients [4], [10]. However, Ajzenberg and colleagues (2009) [11] demonstrated that most European immunocompromised patients with reactivated toxoplasmosis were infected with *T. gondii* clonal type II, whereas clonal type I and non-archetypal *T. gondii* types were isolated from African and South American patients. This suggests that the occurrence of particular *T. gondii* clonal types is influenced by the geographic origin of the patients. Most *T. gondii* isolates obtained in South America, Asia and Africa are genetically distinct from the clonal types I, II and III [12], [13].

*T. gondii* of clonal types I, II and III show different virulence patterns in outbred mice inoculated intraperitoneally (i.p.) with tachyzoites [14], [15]. In this experimental system, *T. gondii* of the clonal types II and III are characterized by LD50 values of ≥10^3^ tachyzoites, i.e. low virulence in mice. By contrast, *T. gondii* isolates of type I are highly virulent for mice with LD100 values of ≤10 tachyzoites [14], [15]. It is not yet clear, whether these differences also imply differences in the pathogenicity of *T. gondii* in humans [15]. There is evidence, however, suggesting that host-genetic factors also contribute to the severity of toxoplasmosis [16], [17], [18], [19], [20], [21].

Several serological assays have been reported that aim at predicting the clonal type of *T. gondii* by which animals or humans are infected [22], [23], [24], [25], [26]. Serotyping is based on the observation that the clonal lineages of *T. gondii* which dominate in North America and Europe differ not only genetically but also in the amino acid sequences of several parasite proteins, leading to polymorphic sites. Antibody responses against these polymorphic sites can thus be allele-specific [22], [27]. Since the three clonal types may have arisen from common ancestors of two closely related but genetically different lineages [8], [28], many of the polymorphic sites are specific for more than one of the three clonal types I, II or III. The pioneering work of Kong et al. (2003) [22] showed that short synthetic peptides derived from polymorphic regions could be used to serologically predict the clonal type of *T. gondii* humans or mice were infected with.

The aim of the present study was to test a panel of sera from *T. gondii* seropositive patients and volunteers (forest workers) from Germany against polymorphic, type-specific sites of 14 *T. gondii* antigens to obtain insights into the clonal types of *T. gondii* these persons were infected with and to explore potential differences in the peptide spectra recognized by patients and seropositive but non-diseased volunteers.

## Materials and Methods

### Patient sera from clinics

In total, 74 *T. gondii* positive human sera were provided by the Institute of Medical Microbiology and Hospital Hygiene, Heinrich-Heine-University, Düsseldorf and the Department of Medical Microbiology and the National Reference Center for Systemic Mycoses, University Medical Center, Göttingen. Out of these, 21 originated from individuals with acute toxoplasmosis, and 53 from individuals with chronic *T. gondii* infection. In addition, these institutions provided 65 samples from serologically *T. gondii*-negative individuals.

Screening of human sera for *T. gondii*-specific immunoglobulin G (IgG) was performed at the institutions providing the sera using an immunofluorescence test (IFT; bioMérieux, Nürtingen, Germany), the LIAISON IgG immunoassay (DiaSorin, Dietzenbach, Germany) or the Mini VIDAS immunoassay system (bioMérieux SA, Marcy l'Etoile, France). *T. gondii*-specific IgM was detected using the Mini VIDAS immunoassay system (bioMérieux SA, Marcy l'Etoile, France), the LIAISON IgM immunoassay (DiaSorin) or the ISAGA IgM immunoassay (bioMerieux). Detailed information about the serological results for each patient serum is shown as supporting information (Table S1). Transient detection of *T. gondii*-specific IgM and eventually IgA was regarded as an indication of an acute infection. In a few patients (n = 7), a persistent IgM response was demonstrated by repeated testing. For these patients, a persistent but inactive (latent) infection was assumed. Presence of IgG and absence of IgM/IgA was regarded as an indication for persistent but inactive (latent) infection.

### Sera from volunteers

A total number of 563 sera were collected from forest workers at all forest offices in the German Federal State Brandenburg [29].

### Ethical considerations

The study reported in our manuscript was a collaborative work of the Toxonet01 project of the National Research Platform for Zoonoses and was approved by the respective ethical committees of the Medical Faculties of the Universities of Düsseldorf (3174, 20/01/09) and Göttingen (8/6/09) and by the State Medical Association of Brandenburg (19/04/10). Serum samples were collected under approved protocols.

For the anonymized patient sera provided by the Institute of Medical Microbiology and Hospital Hygiene, Heinrich-Heine-University, Düsseldorf and the Department of Medical Microbiology and the National Reference Center for Systemic Mycoses, University Medical Center, Göttingen informed consent was obtained verbally, which was in agreement with the ethical committee's approval. All volunteers (forest workers) were included in the study on the basis of written informed consent as described in detail by Mertens et al. (2011) [29].

### Latex agglutination test

A latex agglutination test (LAT, TOXOREAGENT, MAST Diagnostica GmbH, Reinfeld, Germany) was performed according to the instructions of the manufacturer. Results were expressed as reciprocal antibody titres. Sera with reciprocal LAT titres of ≥16 were regarded as seropositive. Reciprocal LAT titres of <16 were considered as seronegative.

### *T. gondii* surface antigen 1 (TgSAG1) immunoblot

Native *T. gondii* surface antigen 1 (TgSAG1) was affinity-purified as previously described [30]. The identity of the purified protein was confirmed using monoclonal antibodies against TgSAG1 (IgG2a P30/3 [ISL, Paignton, UK]). Detection of antibodies against TgSAG1 was performed essentially as described for animal sera [30] with a few modifications. Briefly, human sera were diluted 1∶10 and the conjugate (horse radish peroxidase [HRP] AffiniPure rabbit anti-human IgA+IgG+IgM [H+L], Jackson ImmunoResearch, West Grove, PA, USA) was diluted 1∶500. Reactivity with a protein of a relative molecular mass of 30 kDa was regarded as a *T. gondii* positive reaction. Sera obtained from a LAT positive and a LAT negative volunteer were used as controls.

### Peptides

A total of 54 *T. gondii* synthetic peptides based on amino acid sequences representing polymorphic epitopes of the three archetypal lineages of *T. gondii* were used to detect type-specific antibodies in sera of *T. gondii* seropositive humans from Germany. The respective peptide sequences [22] were derived from 14 *T. gondii* immunogenic proteins, including dense granule proteins, surface antigens and rhoptry proteins (Table S2). Peptide sequences were based on information available for representative *T. gondii* strains of the clonal types I (RH), II (Me49 and Prugniaud) and III (VEG and CEP) (previously described by Kong et al. (2003) [22]). Some of the peptides had sequences specific for more than one of the three clonal lineages. These peptides are referred to as type I–II, type I–III or type II–III.

### Preparation of peptide-microarray slides

Peptides were synthesized and printed on peptide-microarray slides by JPT Peptide Technologies GmbH, Berlin. First, amino-oxy-acetylated peptides were synthesized on cellulose membranes in parallel using the SPOT synthesis technology [31], [32]. After side chain de-protection, the solid phase-bound peptides were transferred into 96-well microtitre filtration plates (Millipore, Bedford, USA) and treated with 200 µl of aqueous triethylamine (0.5% v/v) to cleave the peptides from the cellulose support. Peptide-containing triethylamine solution was filtered off and the solvent removed by evaporation under reduced pressure. The resulting peptide derivatives (50 nmol) were re-dissolved in 25 µl printing solution (70% DMSO, 25% 0.2 M sodium acetate pH 4.5, 5% v/v glycerol) and transferred into 384-well microtitre plates. Two droplets of 0.5 nl peptide solution (1 mM) were deposited per spot on epoxy-functionalized glass slides (Corning Epoxy # 40042; Corning, Lowell, USA) using the non-contact printer Nanoplotter (GESIM, Groβerkmannsdorf, Germany) equipped with a piezoelectric NanoTip (GESIM). The method for chemoselective immobilization on peptide-microarrays was originally described by Panse and colleagues (2004) [33]. This procedure was further optimized for peptide arrays for serum antibody detection and reviewed by Andresen and Grötzinger (2009) [34]. Chicken IgY, cat, human, mouse and pig IgG (Sigma, Munich, Germany and Diatec, Oslo, Norway) were also printed on the slides as antibody controls at a concentration of 500 µg/ml in 100 mM PBS buffer, pH 8.0.

The peptide library was spotted on each slide in triplicate. The slide layout consisted therefore of three identical sub-arrays; peptide and control spots were printed in 21 identical blocks. Printed peptide-microarrays were kept at room temperature for 5 h, washed with de-ionised water, quenched for 1 h with 0.1 mg/ml bovine serum albumin (BSA) in 75 mM saline sodium citrate (SSC) buffer, pH 7.0, containing 0.1% SDS and 750 mM NaCl, at 42°C, washed extensively with 1.5 mM SSC buffer, pH 7.0, followed by washings with de-ionised water and dried using a chip centrifuge (UNIEQUIP Laborgerätebau und Vertriebs GmbH, Planegg, Germany). Resulting peptide-microarrays were stored at 4°C until used.

### Examination of sera by peptide-microarray

Array slides were first incubated with blocking solution (PBS, 0.05% Tween 20, 0.2% I-Block [Applied Biosystems, Bedford, MA, USA]) for 30 min. The slides were then placed into a Microplate Microarray Hardware (Arrayit Corporation, Sunnyvale, CA, USA), which allows to examine arrays separately in a 96 well ELISA format.

Human serum samples (150 µl/well), diluted 1∶200 in blocking solution, were incubated at 37°C for 1 h and washed seven times for 3 min with PBS-T (PBS, pH 7.2; 0.5% Tween 20) on a shaker at room temperature. Conjugate (Cy5-AffiniPure donkey anti-human IgG, Fcγ fragment specific [min X Bov,Hrs,Ms Sr Prot], Jackson ImmunoResearch Laboratories, West Grove, USA) diluted 1∶1000 (1 µg/ml) was added to the wells (150 µl/well), incubated at 37°C for 30 min, and washed as indicated above, followed by three additional washing steps, 1 min each, with sterile-filtered MilliQ water. Afterwards, the slides were spun dry for 10 s using a slide spinner (DW-41MA-230, Qualitron Inc/Eppendorf, Berzdorf, Germany).

### Scanning and measurement of spot signal intensities and data extraction

Peptide-microarray slides were scanned at a wavelength of 635 nm using a GenePix 4000B microarray scanner (Axon Instruments, Concord, Canada) in a low-noise, high-sensitivity photomultiplier tube (PMT) at a level of 100% and a resolution of 10 µm. Images were saved electronically in TIFF and JPG formats.

Image analysis was performed using the circular feature alignment of the GenePix Pro 6.0 software (Axon Instruments) and GenePix Array List (GAL) files. Each circular feature consisted of the peptide spot to determine the foreground and a surrounding area to detect the background reaction. The signals from pixels of each circular feature were used to calculate median net fluorescence intensities of both, the foreground and background of each peptide spot [35], [36].

### Peptide-microarray data analysis

To analyze the raw data (median of signal intensity) in GPR (GenePix Results) files, index values (IVs) were recovered for each peptide-spot as log2 of the quotient of the medians of foreground and background [35], [36]. Each serum was analyzed on a single block with the peptides printed in triplicate in each block. To obtain the serum-specific reaction against each peptide, the means of the IVs for each peptide spot per block (mean sample index value, MSIV) were calculated using the “corrected mean” formula (Microsoft Office EXCEL 2003) to exclude artefacts, i.e. false-positive and -negative signals within the replicas in each block. Application of the “corrected mean” formula had the following effect: If one out of three IVs per sample deviated more than 1.5-fold from the mean of all three IVs, the value was discarded and MSIV was calculated from the two remaining IVs. The peptide-microarrays used in this study failed to meet the criteria required for submission under MIAME based public databases [37], [38]. Therefore MSIV for all sera and peptides are presented as supporting information (Table S3).

To ensure the specificity, we established an individual cut-off for each peptide to classify a reaction with this particular peptide as positive or negative using receiver-operating characteristic (ROC) analysis and the serological status of each serum (Table S3) as a reference standard. The cut-off was selected for each peptide separately using the MSIVs obtained for all *T. gondii* seronegative and seropositive human sera and accepting a maximum of 4% false-positive reactions. The results of ROC analysis (specificity, area under ROC curve, sensitivity and cut-off) for each peptide are shown as supporting information (Table S2). Table S4 shows the results of the application of these cut-offs to the MSIV for each serum and each peptide.

### Statistical analysis

Fisher's exact test and logistic regression were computed with R, version 2.8.1 (R Foundation for Statistical Computing, Vienna, Austria, ISBN 3-900051-07-0, URL http://www.R-project.org) using packages “*Stats*” and “*Epicalc*” respectively [39]. Linear regression and the Wilcoxon rank test were performed using STATISTICA 8 (StatSoft, Tulsa, USA). P-values<0.05 were regarded as statistically significant. Kappa values were calculated using a web-based program (http://www.graphpad.com/quickcalcs/kappa1.cfm). To adjust p-values in multiple testing scenarios, Bonferroni correction was used [40].

To establish cut-offs for each peptide, ROC analysis was applied using the R-package “*DiagnosisMed*”.

The R-package “*vcd*” was used for computing and visualizing log-linear independence models to examine whether reactions with specific peptide cohorts occurred more frequently than with others, i.e. whether the hypothesis of independence had to be rejected. Mosaic plots were used to visualize resulting contingency tables and Pearson residuals. Residuals displayed in mosaic plots represent standardized deviations of observed from expected values calculated by Pearson chi-square. The size of each box within the plot corresponds to the observed frequencies of positive and negative peptide reactions as well as the number of tested peptides within a peptide cohort specific of a clonal type. To present Pearson residuals in mosaic plots, the shading introduced by Friendly et al. (1994) [41] was used. Blue scale shading with a solid blue line (Pearson residuals: >2) or red scale shading with a dashed red line (Pearson residuals: <−2) indicate statistically significant (Pearson chi-squared p-value<0.05) over-, or underrepresentation of certain clonal type-specific peptide reactions within analyzed groups of sera, respectively (rejection of independence hypothesis). Pearson residuals from −2 to 2 are presented by filling the boxes in white colour, presenting the homogeneous distribution of peptide reaction within certain groups.

To perform multiple comparisons of the MSIVs for the tested peptides as well as for clonal type-specific peptide groups, a Post-Hoc-Test (LSD[Least Significant Difference]-Test) on ANOVA results was applied using the R package “*agricolae*”. The differences between the means of positive peptide reactions in the analysis of peptide reactivities within tested groups were regarded as significant if the differences were equal to or higher than the LSD values.

## Results

### Examination for antibodies against *T. gondii* in sera from individuals and seropositive volunteers

**Figure 1:**
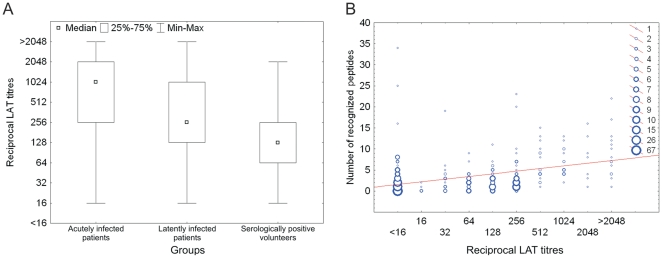
The number of recognized peptides is associated with the titre in LAT (Latex Agglutination Test). Wilcoxon rank test analysis of the *T. gondii* LAT titre distribution within groups of human sera revealed significantly lower LAT titres in sera from volunteers than in sera from latently or in acutely infected individuals (p-value<0.001). No significant differences were observed between sera from acutely and latently infected individuals (A). The association between LAT titre and number of recognized peptides was characterized by an R^2^ value of 0.16 (p-value<0.001) (B).

All sera of *T. gondii*-infected individuals showed reciprocal LAT titres of 16 to >2048 (Table 1; Fig. 1 [A]). The majority of sera from individuals with an acute *T. gondii* infection (15 of 21; 71%) had reciprocal LAT titres of >256, while the majority of sera from individuals with a latent *T. gondii* infection (31 of 53; 59%) showed reciprocal LAT titres of 16 to ≤256. All sera from seronegative patients (n = 65) had reciprocal LAT titres of <16.

**Table 1:** Results in the Latex-Agglutination-Test (LAT) for sera from groups of seropositive and seronegative individuals with acute or latent toxoplasmosis including patients from clinics and volunteers (forest workers).

Group	Infection status	LAT titre									
		<16	16	32	64	128	256	512	1024	2048	>2048
Patients (n = 21)	Acute*		1		1	1	3	4	3	4	4
Patients (n = 53)	Latent#		1	1	7	10	12	3	8	1	10
Volunteers (n = 476)	PositiveΩ		22	44	79	111	95	76	35	9	5
Patients/Volunteers (n = 152)	Negative	152									

* Transient detection of *T. gondii* specific IgM and eventually IgA was regarded as an indication of acute infection.

# Presence of IgG and absence of IgM/IgA was regarded as an indication for persistent but inactive (latent) infection. In a few patients a persistent IgM response was demonstrated by repeated testing. For these patients, a persistent but inactive (latent) infection was assumed.

Ω Antibody isotypes not specified.

The initial LAT screening of a total of 563 sera of forest workers revealed the presence of antibodies against *T. gondii* in the LAT in 476 (84%) (Table 1). The majority of these sera (351 of 476; 74%) had LAT titres ranging between 16 and ≤256 (Fig. 1 [A]). The remaining sera (n = 87) were regarded as seronegative (reciprocal LAT titres <16). Volunteers had significantly lower LAT titres than latently or acutely infected patients (Wilcoxon rank test, p-value<0.001). To confirm the LAT results, all volunteer sera were also tested by TgSAG1 immunoblot. Antibodies to TgSAG1 were detected in 485 of 563 (86%) sera. The agreement between the TgSAG1 immunoblot and the LAT was characterized by a kappa value of 0.913.

Logistic regression analysis revealed that seropositivity in volunteers (forest workers) was positively associated with age in both the LAT and the in-house TgSAG1 immunoblot (LAT: OR 1.09 [95% CI: 1.06–1.12], p_Wald_-value<0.001; TgSAG1 immunoblot: OR 1.07 [95% CI: 1.04–1.1], p_Wald_-value<0.001).

For further examination in the peptide-microarray, 100 volunteer sera which had tested *T. gondii* positive in both assays and 75 volunteer sera with negative results in both *T. gondii* tests were selected randomly. Seropositive and seronegative volunteers were interviewed during sampling to obtain information about their health status. None of the volunteers included in this study reported signs of acute toxoplasmosis.

### Diagnostic specificity and sensitivity of peptide-microarray testing in seronegative and seropositive sera

All sera of seronegative patients and volunteers (n = 140) as well as sera from seropositive patients and volunteers (n = 174) were used to establish peptide-specific cut-offs by ROC analysis. The serological status of patients and volunteers was based on LAT results as a reference standard (Table S3). Application of these cut-offs revealed peptide-dependent diagnostic specificities for the *T. gondii*-negative sera which ranged between 96% and 97% (Table S2). A total of 174 sera, including all seropositive sera from patients (21 with acute and 53 with latent *T. gondii* infection) and 100 randomly selected sera from seropositive volunteers were tested on the peptide-microarray (Table S5). Twenty-two of these 174 (12.6%) seropositive sera failed to recognize any of the 54 peptides. All non-reactive sera had low LAT titres, i.e. showed reciprocal LAT titres between 16 and ≤256.

Sera of patients with an acute *T. gondii* infection recognized a significantly higher number of peptides than the sera of seropositive volunteers (Wilcoxon Rank Test, p-value = 0.012). Also sera of patients with a latent *T. gondii* infection reacted with a statistically significantly higher number of peptides than the sera of seropositive volunteers (Wilcoxon Rank test, p-value = 0.017). The differences between individuals with acute and latent *T. gondii* infections were not statistically significant (Wilcoxon rank test, p-value = 0.256). Linear regression analysis revealed that the number of recognized peptides was statistically significantly associated with the log2-transformed reciprocal LAT titres (p-value<0.001). This association was characterized by an R^2^ value of 0.16 (Figure 1[B]).

### Type-specificity of peptide reactions

In total 9396 (54 peptides×174 sera) peptide reactions were possible: 2436 (14 peptides×174 sera) type I-specific, 2436 (14 peptides×174 sera) type II-specific, 1044 (6 peptides×174 sera) type III-specific, 696 (4 peptides×174 sera) type I–II-, 1740 (10 peptides×174 sera) type I–III-, and 1044 (6 peptides×174 sera) type II–III-specific. In total, 731 of 9396 (8%) possible peptide reactions were observed. Positive reactions were predominantly directed against type II-specific and the type II–III-specific peptides (proportions of peptide reactions 14% [336/2436]) and 10% [106/1044], respectively). The positive reactions against type I- (116/2436 [5%]), type III- (41/1044 [4%]), type I–II- (30/696 [4%]) or type I–III-specific (102/1740 [6%]) peptides were underrepresented.

**Figure 2:**
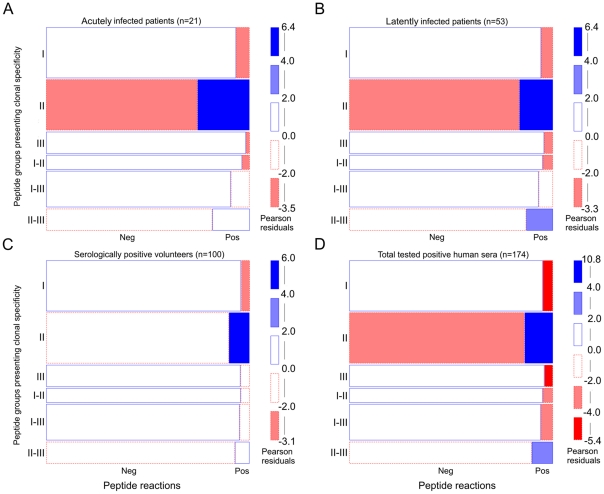
Statistically significant overrepresentation of reactions against clonal type II specific peptides. To determine whether reactions against certain clonal type-specific peptide cohorts (I, II, III, I–II, I–III, or II–III) were over- or underrepresented in various groups of *T. gondii* positive human sera a log-linear model analysis was performed and visualized by mosaic plot: acutely infected individuals (A), latently infected individuals (B) serologically positive volunteers (forest workers) (C) and all tested positive human sera (D). The size of each box corresponds to the observed frequencies of positive (Pos) and negative (Neg) peptide reactions as well as the number of tested peptides within each clonal type-specific peptide cohort. Pearson residuals represent standardized deviations of observed from expected values. The solid blue line indicates that the number of positive or negative reactions is higher than expected but not statistically significant. Blue scale shadings suggest the statistically significant rejection of the null hypothesis, i.e. overrepresentation of certain type-specific peptide reactions (Pearson chi-squared p-value<0.05). Dashed red lines indicate an underrepresentation of positive or negative peptide reactions which is not statistically significant. Red scale shadings suggest a statistically significant rejection of the null hypothesis, i.e. underrepresentation of peptide reactions within tested peptide and human groups (Pearson chi-squared p-value<0.05).

Reactions within clonal type-specific peptide groups were statistically analyzed using a log-linear model. Contingency tables and deviations from independence hypothesis were visualized in mosaic plots (Fig. 2 [A, B, C, D]). Pearson residuals >4 indicated a statistically significant (Chi-squared p-value<0.001) overrepresentation of positive clonal type II-specific peptide reactions within all groups of sera (Fig. 2 [A, B, C, D]). In latently infected patients, type II–III-specific positive peptide reactions were also statistically significantly overrepresented (Pearson residuals: 2–4; Chi-squared p-value<0.05) (Fig. 2 [B]). In patients with an acute infection and in volunteers reactions with type II–III-specific peptides were also overrepresented (white rectangle with solid blue borderline), however, the hypothesis of independence could statistically not be rejected (Fig. 2 [A, C]). Reactions with peptides of type I, III, I–III and I–II were underrepresented in all tested groups of sera (Fig. 2 [A, B, C, D]).

Of 35 dense granule-derived peptides, 7 were recognized by 16%–42% of the sera (Table S5). The majority of these peptides (n = 5) had amino acid sequences specific for type II (dGRA6-II-216(9), GRA3-II-28, GRA6-II-214, dGRA6-II-214, GRA7-II-225; Table S5). The amino acid sequences of the remaining two peptides were specific for both, clonal types I and III (GRA3-I/III-28) or had a sequence specific for clonal type I (NTP3-I-99). Two other dense granule peptides were recognized by 10–11% of the sera. One of these peptides had a type II-specific (dGRA6-II-214(9)) and the other peptide a type I–II-specific (GRA7-I/II-215) amino acid sequence.

Only one of 15 surface antigen-derived (SAG3-II-49) and one of six rhoptry-derived (ROP1-II/III-181) peptides with type II and type II–III specificity were recognized by more than 15% of the sera (Table S5). One rhoptry (ROP1-II/III-359) and none of the remaining surface-derived peptides were among those recognized by 10–15% of the sera.

### Differences in intensity of type-specific peptide reactions

**Figure 3:**
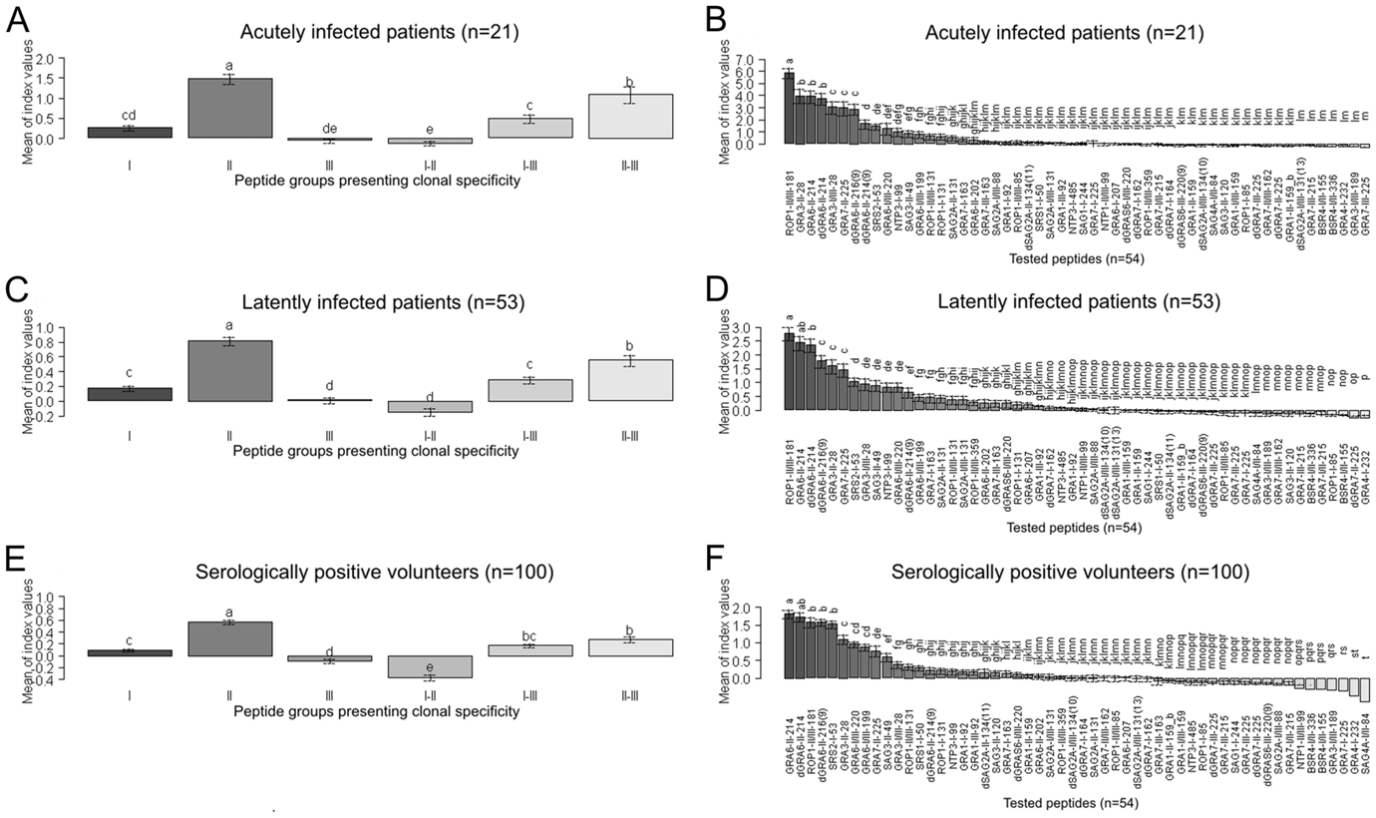
Strongest reaction intensities were recorded for clonal type II specific peptides. To evaluate the intensities (MSIVs) by which single peptides as well as peptide cohorts (I, II, III, I–II, I–III, or II–III) were recognized by *T. gondii* seropositive patient and volunteer groups, ANOVA and the Least Significant Difference (LSD)-Post-Hoc-Test were performed. Whiskers in barplots represent 95% confidence intervals of the means of MSIVs. The differences between the means of MSIVs for single peptides or peptide cohorts within tested groups were regarded as statistically significant, when the differences were equal or higher than the LSD values. Different letters above the whiskers indicate significant differences between the mean intensities in the Post-Hoc-LSD test. Means of MSIVs for each peptide cohort are presented in (A) for the acutely infected patient group (LSD>0.36, p-value<0.05); in (C) for the latently infected patient group (LSD>0.16, p-value<0.05); and in (E) for the seropositive volunteer group (LSD>0.10, p-value<0.05). Means of MSIVs for each single peptide are presented in (B) within the acutely infected patient group (LSD>0.67, p-value<0.05); in (D) within the latently infected patient group (LSD>0.36, p-value<0.05); and in (F) within the seropositive volunteer group (LSD>0.24, p-value<0.05).

To detect differences between reaction intensities (MSIVs) for each tested peptide and for peptide groups presenting clonal type specificity in seropositive patients and volunteers, ANOVA and the LSD-Post-Hoc-Test were performed (Fig. 3). The analyses revealed that for the groups of acutely and latently infected patients, those peptide groups mimicking clonal type II and II–III specificities were recognized by the highest MSIVs as compared to the remaining peptide groups. These differences were statistically significant (LSD>0.36, p-value<0.05 [for acutely infected patients]; LSD>0.16, p-value<0.05 [for latently infected patients]) (Fig. 3 [A, C]). In volunteers, the clonal type II-specific peptide group was also recognized by the highest MSIVs as compared to remaining peptide groups. The difference was statistically significant (LSD>0.102, p-value<0.05 [for seropositive volunteers]) (Fig. 3 [E]).

The intensity of index values was also analysed for each peptide in patient groups with acute or latent *T. gondii* infection and in seropositive volunteers.

Peptides derived from dense granule antigens mimicking type II specificity (GRA6-II-214, dGRA6-II-214, dGRA6-II-216(9)) and one type II–III rhoptry derived peptide (ROP-II/III-181) were detected by the highest MSIVs in all patient and volunteer groups. The differences were statistically significant (LSD>0.67, p-value<0.05 [for acutely infected patients]; LSD>0.36, p-value<0.05 [for latently infected patients]; LSD>0.24, p-value<0.05 [for seropositive volunteers]) (Fig. 3 [B, D, F]).

In patients with acute and latent toxoplasmosis, two further type II-specific peptides (GRA3-II-28, GRA7-II-225) were also detected by the highest MSIVs (LSD>0.67, p-value<0.05 [acutely infected patients]; LSD>0.36, p-value<0.05 [Latently infected patients]) (Fig. 3 [B, D]).

GRA3-I/III-28 and SRS-I-53 peptides also belong to the peptide group recognized by the highest MSIVs in acutely *T. gondii*-infected patients and in seropositive volunteers (LSD>0.67, p-value<0.05 [acutely infected patients]; LSD>0.24, p-value<0.05 [for seropositive volunteers]) (Fig. 3 [B, F]).

### Differences in number of anti-peptide reactions between groups of sera

In all sera from patients and volunteers, reactions against type II dense granule based peptides (GRA3-II-28, GRA6-II-214, GRA7-II-225) and a type II–III rhoptry peptide (ROP1-II/III-181) dominated, i.e. these peptides reacted with more than 20% of the sera (Table S5).

In addition, further peptides were recognized by more than 20% of the sera of patients with acute toxoplasmosis (dGRA6-II-216(9)) but not by those of seropositive volunteers and patients with latent infection. More than 20% of the sera from patients with latent infection recognized a type II surface antigen-derived peptide (SAG3-II-49), but those of volunteers and individuals with acute toxoplasmosis failed to react with this peptide in a proportion of >20%. Two dense granule-based peptides (dGRA6-II-214, GRA3-I/III-28) were recognized by more than 20% of the patients sera (acute, latent), but not by those of seropositive volunteers.

No major differences within each peptide category were observed between the different groups of persons (Table S6), with three exceptions. Individuals with acute toxoplasmosis recognized a significantly higher proportion of peptides with type II-specific sequences than volunteers (p-value = 1.87×10^−11^; Fisher's exact test; Table S6) and latently infected patients, (p-value = 8.24×10^−11^). Latently infected patients recognized a significantly higher proportion of peptides with type II-specific sequences than seropositive volunteers (p-value = 2.98×10^−5^, Fisher's exact test; Table S6). Acutely and latently infected patients recognized a significantly higher proportion of peptides with type II–III specificity as compared to volunteers (p-value = 0.00021, p-value = 0.0036; Fisher's exact test; Table S6).

### Peptide-microarray-based serotyping

Data analysis was carried out to identify the clonal types responsible for the infection of the tested persons and to analyse potential type-specific differences in the peptide spectra recognized by individuals presenting acute or latent *T. gondii* infection and seropositive volunteers.

The majority (n = 124; 71%) of sera showed reactions against synthetic peptides with sequences specific for clonal type II (type II peptides) (Table 2). Forty-two percent (n = 73) or 16% (n = 28) of the sera reacted with type I and type III peptides, respectively, while type II–III, type I–III or I–II peptides were recognized by 49% (n = 85), 36% (n = 62) or 14% (n = 25) sera, respectively.

**Table 2:** Clonal type-specific anti-peptide reactivity of *T. gondii* positive humans.

Peptide specificity	Acute patients (n = 21)		Non-acute patients (n = 53)		Volunteers (forest workers) (n = 100)		Total (n = 174)	
	n	%	n	%	n	%	n	%
I	9	43	26	49	38	38	73	42
II	19	91	38	72	67	67	124	71
III	2	10	8	15	18	18	28	16
I–II	2	10	9	17	14	14	25	14
I–III	15	71	19	36	28	28	62	36
II–III	19	91	30	57	36	3	85	49

Reactions are sorted according to the specificities of peptides.

Based on the anti-peptide reactions, only a fraction of sera could be clearly attributed to either of the three clonal types I, II, or III (Table 3). Among sera that reacted with peptides containing type II specific sequences, 35% (50/142) showed reactions exclusively compatible with clonal type II (Table 3). The remaining 65% (92/142) reacted not only with type II peptides but also with peptides with sequences specific for other clonal types. Most sera reacting with type I and type III peptides could not be clearly assigned to one of the three clonal lineages as many of them also recognized peptides with sequences specific for the other clonal types (Table 3).

**Table 3:** Proportion of human sera showing peptide reactions compatible with *T. gondii* infections by clonal types I, II, or III.

	Sera with anti-peptide reactions exclusively compatible with the respective clonal type				Sera with anti-peptide reactions not exclusively compatible with the respective clonal type			
	Total (%)	AP (%)*	LP (%)*	V (%)*	Total (%)	AP (%)*	LP (%)*	V (%)*
Clonal type I	11 (11)	0 (0)	5 (15)	6 (12)	89 (89)	15 (100)	28 (85)	46 (88)
Clonal type II	50 (35)	5 (25)	16 (36)	29 (37)	92 (65)	15 (75)	28 (64)	49 (63)
Clonal type III	12 (13)	1 (6)	5 (17)	6 (13)	80 (87)	15(94)	25 (83)	40 (87)

Reactions are sorted according to their compatibility with infections of *T. gondii* of the clonal type I, II, or III.

* Data resolved for seropositive patients with acute toxoplasmosis (AP), patients with latent toxoplasmosis (LP) and seropositive volunteers (V).

## Discussion

A number of polymorphic peptides has been described in *T. gondii* antigens which might be suitable to indirectly determine the clonal type of *T. gondii*, humans or mice are infected with [22]. Using such peptides, we tested sera from seropositive volunteers and patients from Germany, to obtain insights into the clonal types of *T. gondii* by which these humans were infected and to examine potential differences in the spectra of peptides recognized by sera of various subgroups.

Several attempts have been made to type *T. gondii* infections by serological techniques using ELISA formats in which synthetic peptides were coupled via keyhole limpet hemocyanin [22], [42] or directly to the solid phase [24], [25], [43], [44]. Others used recombinant antigens for serotyping [23], [26]. We applied a synthetic peptide-microarray format to test a panel of sera simultaneously with all peptides that had previously been used in an ELISA by Kong et al. (2003) [22]. In studies on other infectious diseases, Melnyk et al. (2002) [45] and Mezzasoma et al. (2002) [46] compared peptide-ELISAs with peptide-microarrays and found that peptide-microarrays were much more sensitive than peptide-ELISAs. We therefore expected for the serological typing of *T. gondii* infections that the microarray format should have at least the same sensitivity as the previously reported ELISA format. To ensure a minimum diagnostic specificity of 96% for each peptide, i.e. to make sure that it is unlikely that *T. gondii*-negative humans react with any of these peptides, an individual cut-off was selected for each peptide based on the foreground-background ratio obtained for each peptide and the sera of 140 *T. gondii* seronegative humans.

By conventional techniques, i.e. by PCR-RFLP mediated genotyping using polymorphic loci, we have previously shown that almost all *T. gondii* parasites isolated from cats in Germany showed an allele combination resembling that of clonal type II [47], [48]. Only a single clonal type III isolate and a few isolates with allele combinations different from those of clonal type I, II or III were observed [47]. We therefore expected that the majority of sera from seropositive humans from Germany would recognize peptides with type II-specific amino acid sequences. This turned out to be true since reactions with type II peptides were superior compared to reactions with other peptides in number as well as in intensity. Thus our results are in accord with the results of serotyping studies performed in France and Poland with a limited number (i.e. 8 or 2, respectively) of those 54 peptides we applied (GRA6-II-214, GRA6-I/III-220, dGRA6-II-214, dGRAS6-I/III-220, GRA7-II-225, GRA7-III-225, dGRA7-II-225, dGRA7-III-225) [42], [43]. In addition, our findings confirm the results of studies from Peyron et al. (2006) and Morisset et al. (2009) with recombinant polypeptides mimicking polymorphic clonal type-specific sites of *T. gondii* GRA5 and GRA6 which revealed a significantly dominant clonal type II-specific serological response in patients from France, Italy and Denmark [23], [26].

Although reactions with type II-specific peptides dominated in number and intensity in our study, the sera of many of these humans reacted also with a few peptides with sequences specific for other clonal types. As it is unlikely that all these individuals experienced mixed infections or infections with atypical *T. gondii*, these conflicting results were probably due to the limited specificity of some of the peptides used in serotyping. In these cases, the clonal type of *T. gondii* the affected persons were infected with could not be unambiguously determined. One reason for a low discriminatory power of individual peptides might be the presence of at least one further epitope in the non-polymorphic part of the peptide in addition to the type-specific epitope in the polymorphic site [22], [23]. Therefore, our results suggest that a large panel of well characterized human sera is needed to determine the specificity of each polymorphic peptide. The peptides that are finally used to differentiate clonal type-specific antibody reactions in individuals must be selected extremely carefully. Unfortunately, well-characterized human sera suitable for the evaluation of peptides are rare.

The results of this study also show that the sensitivity by which peptides were recognized varied considerably between the examined groups of patients or volunteers, respectively. For instance, individual type II-specific peptides were recognized by 1% to 42% of the sera.

Each individual serum recognized an almost unique spectrum of peptides. This may reflect an individual maturation of particular plasma cells leading to an increased affinity of the antibodies they produce against different antigens or epitopes of *T. gondii*. The variation in the sensitivity of peptide recognition by different groups of infected persons may be further influenced by a variety of variables, e.g. host-genetic factors, route of infection, secondary infections and time of primary infection [17], [18], [49].

We also found a statistically significant association between the LAT titre and the number of recognized peptides, with the LAT titres explaining 16% of the variability in the number of recognized peptides. Consequently, groups of humans showing differences in mean LAT titres showed similar differences in the number of peptides recognized in the microarray analysis. For instance, sera of patients with an acute *T. gondii* infection had a significantly higher mean LAT titre (Fig. 1 [A]) and recognized a significantly higher number of peptides than sera of seropositive volunteers.

In this study, 13% of the LAT positive sera did not react with any of the 54 peptides used. A previous study, in which two ELISAs with peptides presenting clonal type II and I–III specificity (GRA6-II-214, GRA6-I/III-220) were used, revealed that more than 30% of seropositive sera from Europe (France and Portugal) failed to react in these peptide ELISAs [43]. Sousa and colleagues (2009) suggested that the use of single peptides for serotyping could lead to mistyping. To overcome this problem, a large pool of polymorphic peptides from different antigens should be used [24]. Although we applied a much higher number of peptides as compared to these previous studies, we also observed a high proportion of sera that reacted only with a low number of peptides.

Individual peptides were only recognized by a limited number of sera. GRA6-II-214, for example, has previously been used in a number of other typing studies [22], [24], [25], [41]. This peptide was recognized only by 31% of all tested *T. gondii* antibody positive sera. The truncated variations of this peptide (dGRA6-II-214; dGRA6-II-214(9); dGRA6-II-216(9)) were recognized by even lower proportions of *T. gondii* antibody-positive sera (19%, 10% and 18%, respectively). Therefore, the results of our study show that the sensitivity of individual peptides might be low and, consequently, allow to conclude that serotyping with synthetic peptides requires a large number of highly specific polymorphic peptides.

Our results clearly showed that peptides derived from dense granule proteins, i.e. GRA3, GRA6, and GRA7, were the most reactive ones when tested with human sera. Of 35 dense granule-derived peptides, 7 were recognized by more than 15% of the examined human sera. None of the 15 surface antigen-derived peptides and only 1 of 6 rhoptry antigen-derived peptides rendered a similar result. This finding is in accord with the high potential of dense granule proteins as diagnostic antigens [50], [51], [52], [53].

In our study, a higher proportion of acutely infected patients recognized GRA6 and GRA3 derived peptides as compared to individuals with latent *T. gondii* infection (Table S5). This is in accord with previous results of others who showed that it is possible to discriminate between acute and chronic *T. gondii* infections by using recombinant GRA6 or GRA7 [54], [55], [56].

In conclusion, the results of this study demonstrate that a peptide-microarray assay can be used to detect *T. gondii* clonal type-specific antibody responses in seropositive humans. A previous study suggested that individuals in the study area were mainly exposed to clonal type II *T. gondii* [47], [48]. Indeed, positive peptide reactions presenting clonal type II specificity were statistically significantly overrepresented in the tested human population and the intensity by which type II peptides were recognized was significantly higher than the intensity by which peptides with other specificities were detected. However, to establish serotyping assays with higher resolution, well-characterized reference sera and further specific peptide markers are needed.

## Supporting Information

Table S1 **Detailed serological results for patient sera.**
(XLS)

Table S2 **Peptides with clonal type specific amino-acid sequences used for typing the anti-** ***T. gondii*** **IgG response in humans.**
(DOC)

Table S3 **Corrected mean sample index values (signal intensity) listed for all peptides and sera.**
(XLS)

Table S4 **Serum-peptide reactions as determined by peptide specific cut-offs for all tested peptides and sera.** Positive reactions are signed as original index value and negative reactions signed as “0”.
(XLS)

Table S5 **Number of sera from seropositive patients and volunteers (forest workers) recognizing peptides with clonal type-specific amino acid sequences.**
(DOC)

Table S6 **Statistical analysis (Fisher's exact test) of differences in the proportion of peptides recognized by different groups of toxoplasmosis patients (acute, latent) or seropositive volunteers (forest workers).**
(DOC)
