# Supplementary material for: Analysis of Clonal Type-Specific Antibody Reactions in Toxoplasma gondii Seropositive Humans from Germany by Peptide-Microarray
Source: PLoS One. 2012 Mar 28;7(3):e34212. doi: 10.1371/journal.pone.0034212 (PMC3314601; doi:10.1371/journal.pone.0034212)
Supplement: Table S2 — Peptides with clonal type specific amino-acid sequences used for typing the anti- T. gondii IgG response in humans. (DOC) [file pone.0034212.s002.doc]

**Table S2.** Peptides with clonal type specific amino-acid sequences used for typing the anti-*T. gondii* IgG response in humans.

| Peptide name | Peptide amino-acid sequence | Clonal type^*^ | Expected reactivity ^#^ | Cut-off value | Area under ROC curve (AUC) | Sensitivity in sero-positive human sera (%) | Specificity in sero-negative human sera (%) |
| --- | --- | --- | --- | --- | --- | --- | --- |
| ROP1-I-85 | PVRGPDQVPAC | I | yes | 1.35 | 0.56 | 1 | 96 |
| GRA7-I-225 | CVPESGEDGEDARQ | I | yes | 0.81 | 0.55 | 7 | 96 |
| GRA7-I-163 | ELTEEQQRGDEPLC | I | yes | 3.14 | 0.51 | 3 | 96 |
| dGRA7-I-162 | CPELTEEQQRG | I | yes | 1.24 | 0.50 | 6 | 96 |
| dGRA7-I-164 | C——LTEEQQRG | I | yes | 1.30 | 0.53 | 4 | 97 |
| GRA6-I-207 | CGRGEGGAEDDRRP | I | yes | 0.99 | 0.51 | 6 | 96 |
| GRA6-II-214 | CLHPGSVNEFDF | II | yes | 2.75 | 0.74 | 31 | 96 |
| dGRA6-II-214 | CLHPGSVNEFD | II | yes | 3.60 | 0.74 | 19 | 96 |
| dGRA6-II-216(9) | C—PGSVNEFDF | II | yes | 2.81 | 0.64 | 18 | 96 |
| dGRA6-II-214(9) | CLHPGSVNE | II | yes | 2.24 | 0.59 | 10 | 96 |
| GRA3-II-28 | ADQPGNHQALAEPVC | II | yes | 1.87 | 0.78 | 31 | 96 |
| GRA7-II-225 | CVPESGKDGEDARQ | II | yes | 1.22 | 0.55 | 42 | 97 |
| dGRA7-II-225 | CVPESGKDGEDA | II | yes | 1.50 | 0.53 | 2 | 96 |
| SAG2A-II-131 | PAGRNNDGGSSAPTPKC | II | yes | 1.90 | 0.63 | 5 | 96 |
| dSAG2A-II-134(11) | RNNDGGSSAPC | II | yes | 1.74 | 0.50 | 5 | 96 |
| GRA6-II-202 | CGRGEGG—EDDRRPL | II | yes | 1.55 | 0.52 | 5 | 96 |
| SAG3-II-49 | GNSRRKITYC | II | yes | 1.73 | 0.67 | 17 | 96 |
| GRA7-III-225 | CVPESGEDREDARQ | III | yes | 1.25 | 0.54 | 6 | 96 |
| GRA7-III-215 | CSRQPAPEHEVPES | III | yes | 1.07 | 0.53 | 6 | 96 |
| GRA7-III-163 | ELTEQQQTGDEPLC | III | yes | 2.09 | 0.50 | 4 | 96 |
| dGRA7-III-225 | CVPESGEDREDA | III | yes | 1.21 | 0.51 | 4 | 96 |
| dGRAS6-III-220(9) | CLHPERVNV | III | yes | 1.24 | 0.52 | 1 | 96 |
| GRA7-I/II-215 | CSRQPALEQEVPES | I and II | yes | 0.72 | 0.53 | 11 | 96 |
| GRA6-I/III-220 | CLHPERVNVFDY | I and III | yes | 2.53 | 0.53 | 7 | 96 |
| dGRAS6-I/III-220 | CLHPERVNVFD | I and III | yes | 1.35 | 0.54 | 3 | 96 |
| GRA3-I/III-28 | ADQPENHQALAEPVC | I and III | yes | 1.92 | 0.72 | 19 | 97 |
| SAG2A-I/III-131 | PAGRNND—GSSAPTPKC | I and III | yes | 2.03 | 0.59 | 4 | 96 |
| dSAG2A-I/III-131(13) | PAGRNND—GSSAPC | I and III | yes | 1.82 | 0.51 | 3 | 96 |
| dSAG2A-I/III-134(10) | RNND—GSSAPC | I and III | yes | 1.38 | 0.52 | 3 | 96 |
| GRA6-I/III-199 | CGNEGRGYGGRGEG | I and III | yes | 2.61 | 0.56 | 5 | 96 |
| ROP1-II/III-85 | PVRDPRQVPGRGEC | II and III | yes | 1.71 | 0.53 | 4 | 96 |
| ROP1-II/III-359 | CTRVRGALR—GRGR | II and III | yes | 0.58 | 0.61 | 13 | 96 |
| GRA7-II/III-162 | CPELTEQQQTG | II and III | yes | 1.75 | 0.52 | 1 | 96 |
| NTP3-I-99 | SIQLIGAGKRFAGLRC | I | no | 1.18 | 0.65 | 16 | 96 |
| NTP3-I-485 | CAPMIVTGGGMLAAINT | I | no | 1.63 | 0.63 | 1 | 96 |
| SAG1-I-244 | SDKGATLTIKKEAFPC | I | no | 1.56 | 0.58 | 2 | 96 |
| SRS1-I-50 | SMTSPLLTWDGNKVTC | I | no | 1.84 | 0.51 | 3 | 96 |
| SRS2-I-53 | GPPYRYEPEKFTC | I | no | 3.45 | 0.53 | 4 | 96 |
| GRA1-I-92 | CSYSEVGNVNVEE | I | no | 1.60 | 0.50 | 3 | 96 |
| GRA4-I-232 | CTEDSGLTGVKDSSS | I | no | 0.74 | 0.51 | 3 | 96 |
| ROP1-I-131 | NSEDDDTFHDAC | I | no | 2.43 | 0.54 | 5 | 96 |
| SAG3-II-120 | CHIDAKDQDD | II | no | 2.00 | 0.52 | 1 | 96 |
| GRA1-II-159 | CQDEMNVIDDVQQ | II | no | 1.00 | 0.54 | 3 | 96 |
| GRA1-II-159_b | SAAIGGRMVSRTLRDNIPGC | II | no | 0.69 | 0.54 | 4 | 96 |
| GRA1-III-92 | CSYSEVGDVNVEE | III | no | 1.75 | 0.50 | 2 | 96 |
| BSR4-I/II-155 | KVNEQREESNKSQKC | I and II | no | 1.10 | 0.53 | 3 | 96 |
| BSR4-I/II-336 | PKKDKESGTETGAPC | I and II | no | 1.47 | 0.54 | 2 | 96 |
| SAG4A-I/II-84 | CKDEPVELAAL | I and II | no | 1.69 | 0.60 | 1 | 96 |
| SAG2A-I/III-88 | PGAVLTAKVQQPAKGPC | I and III | no | 0.93 | 0.51 | 5 | 96 |
| GRA1-I/III-159 | CQDEMKVIDDVQQ | I and III | no | 1.12 | 0.52 | 2 | 96 |
| GRA3-I/III-189 | RRKPKDEGAGVDKAC | I and III | no | 0.64 | 0.54 | 6 | 96 |
| NTP1-II/III-99 | SIRLIREGKRFTGLRC | II and III | no | 1.13 | 0.53 | 4 | 96 |
| ROP1-II/III-131 | NSEDD—TFHDAC | II and III | no | 2.55 | 0.51 | 3 | 96 |
| ROP1-II/III-181 | QELPPPNAQELC | II and III | no | 2.66 | 0.85 | 35 | 97 |

Peptides were described and validated by Kong et al. (2003).

^*^ I, II and III represent polymorphisms in peptide sequences, which are specific for the three archetypal lineages.

^#^ Peptides which did not react with human sera when validated by Kong et al. (2003) are indicated by “no”. Those reacting are indicated by “yes”.
