# Supplementary material for: Analysis of Clonal Type-Specific Antibody Reactions in Toxoplasma gondii Seropositive Humans from Germany by Peptide-Microarray
Source: PLoS One. 2012 Mar 28;7(3):e34212. doi: 10.1371/journal.pone.0034212 (PMC3314601; doi:10.1371/journal.pone.0034212)
Supplement: Table S5 — Number of sera from seropositive patients and volunteers (forest workers) recognizing peptides with clonal type-specific amino acid sequences. (DOC) [file pone.0034212.s005.doc]

**Table S5.** Number of sera from seropositive patients and volunteers (forest workers) recognizing peptides with clonal type-specific amino acid sequences.

|  | Patients, acute*  (n = 21) | | Patients, latent*  (n = 53) | | Volunteers*  (n = 100) | | Total positive  (n = 174) | |
| --- | --- | --- | --- | --- | --- | --- | --- | --- |
| Peptides | n | % | n | % | n | % | n | % |
| ROP1-I-85 | 1 | 5 | 0 | 0 | 1 | 1 | 2 | 1 |
| GRA6-I-207 | 1 | 5 | 5 | 9 | 5 | 5 | 11 | 6 |
| GRA7-I-225 | 2 | 10 | 5 | 9 | 6 | 6 | 13 | 7 |
| GRA7-I-163 | 1 | 5 | 3 | 6 | 2 | 2 | 6 | 3 |
| dGRA7-I-162 | 0 | 0 | 4 | 8 | 7 | 7 | 11 | 6 |
| dGRA7-I-164 | 0 | 0 | 1 | 2 | 6 | 6 | 7 | 4 |
| ROP1-I-131 | 3 | 14 | 2 | 4 | 3 | 3 | 8 | 5 |
| GRA1-I-92 | 1 | 5 | 1 | 2 | 4 | 4 | 6 | 3 |
| GRA4-I-232 | 1 | 5 | 1 | 2 | 4 | 4 | 6 | 3 |
| SRS2-I-53 | 0 | 0 | 2 | 4 | 5 | 5 | 7 | 4 |
| SRS1-I-50 | 1 | 5 | 2 | 4 | 3 | 3 | 6 | 3 |
| SAG1-I-244 | 1 | 5 | 1 | 2 | 2 | 2 | 4 | 2 |
| NTP3-I-485 | 0 | 0 | 1 | 2 | 0 | 0 | 1 | 1 |
| NTP3-I-99 | 7 | 33 | 13 | 25 | 8 | 8 | 28 | 16 |
| dGRA6-II-216(9) © | 9 | 43 | 13 | 25 | 10 | 10 | 32 | 18 |
| GRA3-II-28 © | 15 | 71 | 17 | 32 | 23 | 23 | 55 | 31 |
| GRA6-II-214 © | 15 | 71 | 19 | 36 | 20 | 20 | 54 | 31 |
| dGRA6-II-214(9) © | 6 | 29 | 6 | 11 | 5 | 5 | 17 | 10 |
| dGRA6-II-214 © | 9 | 43 | 15 | 28 | 9 | 9 | 33 | 19 |
| dSAG2A-II-134(11) | 0 | 0 | 1 | 2 | 7 | 7 | 8 | 5 |
| SAG2A-II-131 | 2 | 10 | 3 | 6 | 3 | 3 | 8 | 5 |
| GRA6-II-202 | 2 | 10 | 4 | 8 | 2 | 2 | 8 | 5 |
| dGRA7-II-225 | 0 | 0 | 1 | 2 | 3 | 3 | 4 | 2 |
| GRA7-II-225 | 13 | 62 | 26 | 49 | 34 | 34 | 73 | 42 |
| SAG3-II-49 | 4 | 19 | 12 | 23 | 14 | 14 | 30 | 17 |
| GRA1-II-159_b | 0 | 0 | 3 | 6 | 4 | 4 | 7 | 4 |
| SAG3-II-120 | 0 | 0 | 0 | 0 | 1 | 1 | 1 | 1 |
| GRA1-II-159 | 0 | 0 | 1 | 2 | 5 | 5 | 6 | 3 |
| GRA7-III-215 | 1 | 5 | 3 | 6 | 7 | 7 | 11 | 6 |
| dGRA7-III-225 | 1 | 5 | 3 | 6 | 3 | 3 | 7 | 4 |
| dGRAS6-III-220(9) | 0 | 0 | 0 | 0 | 1 | 1 | 1 | 1 |
| GRA7-III-225 | 0 | 0 | 3 | 6 | 8 | 8 | 11 | 6 |
| GRA7-III-163 | 0 | 0 | 3 | 6 | 4 | 4 | 7 | 4 |
| GRA1-III-92 | 0 | 0 | 1 | 2 | 3 | 3 | 4 | 2 |
| GRA7-I/II-215 | 2 | 10 | 7 | 13 | 10 | 10 | 19 | 11 |
| SAG4A-I/II-84 | 1 | 5 | 1 | 2 | 0 | 0 | 2 | 1 |
| BSR4-I/II-336 | 0 | 0 | 1 | 2 | 3 | 3 | 4 | 2 |
| BSR4-I/II-155 | 0 | 0 | 1 | 2 | 4 | 4 | 5 | 3 |
| GRA3-I/III-28 © | 13 | 62 | 11 | 21 | 9 | 9 | 33 | 19 |
| GRA1-I/III-159 | 0 | 0 | 1 | 2 | 3 | 3 | 4 | 2 |
| GRA3-I/III-189 | 0 | 0 | 2 | 4 | 9 | 9 | 11 | 6 |
| SAG2A-I/III-131 | 0 | 0 | 4 | 8 | 3 | 3 | 7 | 4 |
| GRA6-I/III-220 | 3 | 14 | 5 | 9 | 4 | 4 | 12 | 7 |
| dSAG2A-I/III-131(13) | 0 | 0 | 3 | 6 | 3 | 3 | 6 | 3 |
| dGRAS6-I/III-220 | 0 | 0 | 4 | 8 | 2 | 2 | 6 | 3 |
| dSAG2A-I/III-134(10) | 0 | 0 | 2 | 4 | 4 | 4 | 6 | 3 |
| GRA6-I/III-199 | 2 | 10 | 2 | 4 | 4 | 4 | 8 | 5 |
| SAG2A-I/III-88 | 1 | 5 | 2 | 4 | 6 | 6 | 9 | 5 |
| ROP1-II/III-85 | 1 | 5 | 1 | 2 | 5 | 5 | 7 | 4 |
| ROP1-II/III-359 | 1 | 5 | 12 | 23 | 10 | 10 | 23 | 13 |
| GRA7-II/III-162 | 0 | 0 | 0 | 0 | 2 | 2 | 2 | 1 |
| ROP1-II/III-181 ^©, #, £^ | 19 | 90 | 24 | 45 | 18 | 18 | 61 | 35 |
| ROP1-II/III-131 | 2 | 10 | 2 | 4 | 2 | 2 | 6 | 3 |
| NTP1-II/III-99 | 0 | 0 | 2 | 4 | 5 | 5 | 7 | 4 |

* Data resolved for seropositive patients with acute toxoplasmosis, seropositive patients with latent toxoplasmosis and seropositive volunteers

© Patients with acute toxoplasmosis recognized statistically significantly (p-value < 0.00089, Fisher’s exact test) more often this peptide than volunteers.

^#^ Patients with acute toxoplasmosis recognized statistically significantly (p-value < 0.00089, Fisher’s exact test) more often this peptide than latent infected patients.

^£^ Patients with latent toxoplasmosis recognized statistically significantly (p-value < 0.00089, Fisher’s exact test) more often this peptide than volunteers.
