# Supplementary material for: Analysis of Clonal Type-Specific Antibody Reactions in Toxoplasma gondii Seropositive Humans from Germany by Peptide-Microarray
Source: PLoS One. 2012 Mar 28;7(3):e34212. doi: 10.1371/journal.pone.0034212 (PMC3314601; doi:10.1371/journal.pone.0034212)
Supplement: Table S6 — Statistical analysis (Fisher's exact test) of differences in the proportion of peptides recognized by different groups of toxoplasmosis patients (acute, latent) or seropositive volunteers (forest workers). (DOC) [file pone.0034212.s006.doc]

**Table S6.** Results of a statistical analysis (p-values, Fisher’s exact test) of differences in the proportion of peptides with type specific sequences recognized by different groups of toxoplasmosis patients (acute, latent) or seropositive volunteers (forest workers).

| Groups of humans compared | Clonal type | | | | | |
| --- | --- | --- | --- | --- | --- | --- |
|  | I | II | III | I-II | I-III | II-III |
| Acute *vs* latent | 0.55 | 8.24E-04* | 0.25 | 1 | 0.28 | 0,17 |
| Acute *vs* volunteers | 0.084 | 1.87E-11* | 0.2 | 1 | 0.018 | 0,00021* |
| Latent *vs* volunteers | 0.12 | 2.98E-05* | 1 | 0.8 | 0.09 | 0,0036* |

*Statistically significant
